# Long-Term Health Effects of COVID-19 in Tunisia, 2020–2021

**DOI:** 10.3390/ijerph23010049

**Published:** 2025-12-30

**Authors:** Sonia Dhaouadi, Hind Bouguerra, Aicha Hechaichi, Hejer Letaief, Mouna Safer, Chaima Aichouch, Amenallah Zouayti, Myriam Bougatef, Arwa Neffati, Nawel El Mili, Rim Mhadhbi, Nissaf Bouafif ép Ben Alaya

**Affiliations:** 1 National Observatory of New and Emerging Diseases, Tunis 1002, Tunisianissafba@yahoo.fr (N.B.é.B.A.); 2 LR01ES04 Epidémiologie et Prévention des Maladies Cardiovasculaires en Tunisie, Faculty of Medicine of Tunis, University Tunis El Manar, Tunis 1007, Tunisia; 3 Mediterranean and Black Sea Programme in Intervention Epidemiology Training (MediPIET), European Centre for Disease Prevention and Control (ECDC), Solna, 16973 Stockholm, Sweden

**Keywords:** long COVID, SARS-CoV-2, COVID-19, prevalence, determinants, Tunisia, epidemiology

## Abstract

**Background:** Some patients suffer from persistent symptoms following a COVID-19 infection, referred to as long COVID. The aims of the study were to estimate the prevalence of long COVID and study its determinants in Tunisia. **Methods:** We conducted a nationwide cross-sectional study among a representative sample of COVID-19 survivors residing in Tunisia between June and August 2022. We selected a random sample, stratified by age and region, among residents registered in the national surveillance database with a SARS-CoV-2 positive test taken from September 2020 to September 2021 (*n* = 479,743). The expected sample size was 384. We defined a patient with long COVID as having at least one self-reported symptom persisting for more than four weeks after the first confirmation of SARS-CoV-2 infection (RT-PCR or Ag-RDT) and not explained by an alternative diagnosis. Trained healthcare workers interviewed consenting respondents by phone using a structured questionnaire. We described continuous variables using median and interquartile range (IQR). We measured the prevalence of long COVID and its 95% confidence interval (95% CI). We estimated the association between explanatory variables (socio-demographic, lifestyle and comorbidities, SARS-CoV-2 history infection, COVID-19 vaccination status) and long COVID using a log-binomial model, reporting adjusted prevalence ratios (a-PR) and its 95% CI. **Results:** Of 1094 persons contacted, 416 were enrolled (response rate: 38%). Long-COVID prevalence was 64% (267/416); 95% CI [59–69%]. The sex ratio (M:F) was 0.72. Age ranged from 1 to 101 years, with a median of 41 years (IQR:31–55 years). The most common symptoms were fatigue (63%), myalgia/arthralgia (33%), and cognitive symptoms (52%). Median duration of long-COVID symptoms was 11 months (IQR: 3–14 months). In multivariate analysis, experiencing acute COVID-19 (a-PR = 1.5; 95% CI [1.0–2.1]), being a woman of childbearing age (a-PR = 1.2; 95% CI [1.0–1.4]) and residing in the central region (a-PR = 1.5; 95% CI [1.1–2.0]) were significantly associated with a higher prevalence of long COVID. **Conclusions:** Long COVID is prevalent in Tunisia affecting patients with multiple symptoms initially, those residing in the central region and young women. We recommend to enhance healthcare access and medical follow-up both during and after the infection, focusing on identified risk groups. We also recommend to conduct further research to optimize management of long-COVID patients.

## 1. Introduction

Severe acute respiratory syndrome coronavirus 2 (SARS-CoV-2) infection, commonly known as COVID-19, resulted in a worldwide pandemic with substantial mortality and morbidity. The clinical presentation of COVID-19 was variable; according to the initial report from the Chinese Center for Disease Control and Prevention in February 2020, approximately 81% of individuals infected with SARS-CoV-2 developed mild to moderate disease, while 14% developed severe disease requiring hospitalization and 5% developed critical illness (death) [1]. The average recovery period from COVID-19 ranged from 2 to 3 weeks depending on symptoms severity [2]. While most infected people with SARS-CoV-2 recovered within a few days to a couple of weeks after the initial infection, 10–20% of those infected experienced persistent symptoms following their recovery, which is commonly referred to as long COVID [3]. According to the Global Burden of Disease (GBD) study in 2020 and 2021, an estimated 144.7 million (95% UI [54.8–312.9]) people worldwide suffered from long COVID. This accounts for approximately 3.69% (1.38–7.96) of the total reported COVID-19 cases [4].

Long COVID is an emerging, complex, heterogeneous, and challenging medical condition. Although acute COVID-19 mainly affects the respiratory system, long-COVID symptoms can involve the cardio-vascular system, kidneys, liver, muscles, skin, nervous system, and others organs [5]. According to the Centers for Disease Control and Prevention (CDC), long-COVID symptoms are a wide range of new, returning, or persistent health problems that people experience after being infected with the virus, lasting for a minimum of four weeks after the initial infection [6]. On the other hand, according to the World Health Organization (WHO), long COVID occurs in individuals with a history of probable or confirmed SARS-CoV-2 infection, usually three months from the onset of COVID-19 with symptoms that persist for at least two months and cannot be explained by an alternative diagnosis [3]. The prevalence of long COVID is very heterogeneous and varies among regions worldwide [7,8]. Additionally, there is limited knowledge regarding the potential predictors of long-COVID symptoms and complications, especially in healthy individuals who had recovered from COVID-19. In Tunisia, as of September 2021, the cumulative number of reported COVID-19 cases and deaths were, respectively, 736,332 and 25,202 [9]. Few sub-national studies have attempted to estimate the prevalence of long COVID starting from 2021. According to a multicentric TUN-EndCOV study conducted between 20 January and 10 May 2021, the prevalence of long COVID was 77% [10]. Another study conducted from April to July 2021 among out-patients presenting to an emergency department of a reference hospital for respiratory diseases revealed a prevalence of 64% [11]. The third study, carried out in February 2022 among a convenience sample using an online self-administered questionnaire, reported a long COVID prevalence of 46% [12].

The aim of this study was to estimate the prevalence of long COVID among a representative nationwide sample and to identify its determinants in Tunisia. The secondary aim was to describe the prevalence and the median duration of long-COVID symptoms.

## 2. Materials and Methods

### 2.1. Study Design, Period, and Population

We conducted an analytical cross-sectional study among a representative sample of COVID-19 cases diagnosed in Tunisia between September 2020 and September 2021. We performed this study at the National Observatory of New and Emerging Diseases (ONMNE) during June–August 2022.

#### 2.1.1. Inclusion Criteria

We recruited COVID-19 survivors (symptomatic and asymptomatic during COVID-19 acute phase) who were registered in the national surveillance database and had tested positive for SARS-CoV-2 using either the RT-PCR or Antigen Rapid Diagnostic Test (Ag-RDT) between September 2020 to September 2021 according to the national testing strategy, with available telephone number and consented to participate in this study. Overall, there were 479,743 eligible individuals registered in the COVID-19 surveillance database.

#### 2.1.2. Exclusion Criteria

We excluded non respondents and non-consenting individuals.

### 2.2. Sampling

**Figure 1:**
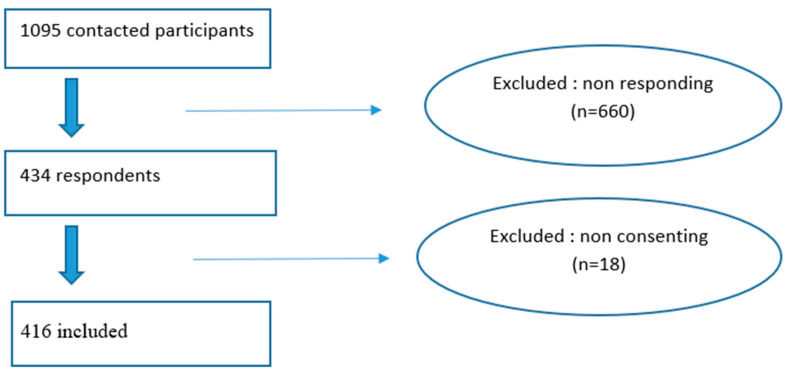
Data flow diagram of selected participants, long-COVID cross-sectional study, Tunisia, 2020/2021.

In order to ensure representativeness of our sample, we performed a stratified random sampling with probability proportional to size (PPS) using the following strata: age groups (years): <18; 18–64; ≥65 and region of residence: North; Center and South (Figure 1). The sampling frame was the national COVID-19 surveillance database, which included all surviving SARS-CoV-2-infected individuals who were notified to the ONMNE from September 2020 to September 2021 with available telephone number (*n* = 479,743).

We used Open epi software (version 3.01, 2013) for the sample size calculation following this formula [13]:

*n* = [DEFF × Np(1 − p)]/[(d^2^/Z^2^ (1 − α/2) × (N − 1) + p × 1 − p)]; with:

p: expected frequency (prevalence) = 50%

d: absolute precision = 5%,

DEFF: design effect = 1,

α: alpha = 5%,

Z^2^ (1 − α/2) = 1.96^2^.

*N*: population size = 479,743

The expected sample size was 384 (without adjustment on non-response rate) and 768 after adjustment on non-response rate by telephone (50%). Following the proportional distribution of COVID-19 cases by age and residence in the national database, the sample size required by strata is as shown in Table 1.

### 2.3. Operational Definitions

#### 2.3.1. Long COVID

We defined long COVID as having at least one self-reported symptom persisting for four weeks or more following the initial confirmed SARS-CoV-2 infection (RT-PCR or Ag-RDT) and not explained by an alternative diagnosis. We used an adapted definition between CDC and WHO guidelines [3,6].

#### 2.3.2. Severe COVID-19

We defined severe COVID-19 according to the national guidelines, as patients who developed complications during the acute phase of illness and required one of the following interventions: oxygen therapy, hospitalization or intensive care [14]. We classified all the other cases including asymptomatic, mild, and moderate cases as non-severe.

#### 2.3.3. Intense COVID-19

We defined intense COVID-19 as patients who developed at least four symptoms (any symptoms of the acute phase) during the acute phase of COVID-19 illness. We used this definition based on the symptoms number given the absence of validated classification of COVID-19 intensity and existing classifications focus on disease severity rather than symptom burden and disease intensity.

#### 2.3.4. SARS-CoV-2 Reinfection

We defined SARS-CoV-2 reinfection as patients tested positive for SARS-CoV-2 more than 60 days after their previous confirmed infection according to the national guidelines [15].

#### 2.3.5. COVID-19 Vaccination

We defined COVID-19 vaccinated as patients who received at least one dose of an approved COVID-19 vaccine according to the national vaccination schedule [16].

#### 2.3.6. SARS-CoV-2 Dominant Variants

We divided the study population based on their date of diagnosis into three epidemic periods according to the circulation of SARS-CoV-2 variant of concern (VOC): [17,18]

1. 1 September 2020–16 January 2021—wild strain
2. 17 January 2021–26 June 2021—circulation of Alpha VOC
3. 27 June 2021–30 September 2021—circulation of Delta VOC

### 2.4. Data Collection

Before data collection, interviewers participated in training seminars at the ONMNE covering the study protocol, questionnaire administration, and ethical considerations. Trained healthcare workers in this purpose interviewed consented respondents by telephone (15–20 min) using a structured close-ended questionnaire in the Arabic language (Tunisian Arabic dialectic) which included 52 questions divided into five items (Supplementary Materials S1):

- Participant’s socio-demographic characteristics;
- Concomitant disease and lifestyle (tobacco and alcohol consumption, obesity and sedentary lifestyle);
- COVID-19 vaccination status (number and date of vaccination);
- COVID-19 medical history (date and mode of confirmation, symptoms duration and severity of acute SARS-CoV-2 infection, number of SARS-CoV-2 infection, COVID-19 treatment);
- Long-COVID symptoms (types and duration);
- Non-response was defined after two unsuccessful contact attempts.

### 2.5. Pilot Study

We performed a pilot study on a small sample from the study population before the main study in order to assess the survey’s feasibility, the comprehensiveness of questionnaire items by investigators and participants and to estimate the maximum number of questionnaires that could be effectively administrated by one investigator. After the pilot study, the questionnaire was revised, with a reduction in length and simplification of selected items to improve its suitability for telephone administration.

### 2.6. Data Entry and Analysis

The outcome was long COVID. The explanatory variables were socio-demographic, lifestyle and comorbidities, history of SARS-CoV-2 infection, and COVID-19 vaccination status.

#### 2.6.1. Descriptive Analysis

To assess non-response, we compared selected characteristics between respondents and non-respondents according to age group, gender, region of residence, and period of COVID-19 confirmation using χ^2^ test. A non-responder to a telephone call was defined as non-responding after two consecutive attempts.

We described continuous variables using the mean ± standard deviation or median and interquartile range (IQR: 25–75th percentile), when appropriate. We calculated proportions for the categorical variables. We reported missing data and non-applicable responses for descriptive analyses. Percentages comparisons between groups (participants with and without long COVID) were performed using χ2 test or Fisher exact, as appropriate. The binomial confidence interval (CI) method was used to estimate uncertainties around prevalence of long COVID and symptoms. We used the receiver operating characteristic (ROC) curve to determine the cut-off of quantitative variables (number of symptoms during SARS-CoV-2 acute infection) (Supplementary Materials S2). To assess construct validity, we conducted an exploratory Spearman correlation analysis across long-COVID domain and constructed the heat map (Supplementary Materials S3).

#### 2.6.2. Univariable and Multivariable Analysis

We estimated the crude prevalence ratio (PR) and its 95% confidence intervals (CIs) in invariable analysis to assess the strength of association between long COVID and explanatory variables. For multivariable analysis, we used log-binomial regression to estimate adjusted PR (a PR) and its 95% CI in order to estimate the independent effect of each explanatory variable in long COVID. We included in the initial model all variables in univariable analysis with *p* ≤ 0.2. We checked for multicollinearity by calculating the variance inflation factor (VIF) (VIF > 5 indicates the elimination of variables from the model). We excluded missing data and non-applicable responses from theses analyses. We assessed the model’s fitness by Akaike information criterion (AIC). We retained the final model with minimum variables and lowest value of AIC. For all statistical analyses, we used a *p*-value ≤ 0.05 as degree of significance. We used Epi info (version 7.2.5.0) for the data entry and R software (version 4.1.2) for the data analysis.

### 2.7. Ethical Considerations

This study was approved by the ethical committee of Pasteur Institute of Tunis (IPT) (reference number 2022/16/E). We required oral informed consent before participation from all participants (for minors aged less than 18 years, consent was taken from legal guardian). Data were analyzed anonymously. We ensured data confidentiality during all the steps of study.

## 3. Results

Out of the 1094 participants who were contacted, 416 responded to the survey resulting in a response rate of 38%, as shown in the data flow diagram (Figure 1).

We checked the representativeness of our sample compared to the source population according to region and age group in Supplementary Materials S4 (*p* = 0.8 and 0.4 respectively).

The non-response analysis showed no significant difference on characteristics of respondents and non-respondents (Table 2).

### 3.1. Study Population Characteristics

The sex ratio (M:F) was 0.96. The median age was 41 years with an IQR: 30–56. One hundred thirty-nine (33%) respondents were women of childbearing age and 44 (11%) were healthcare workers (HCW). One third (34%) of respondents reported having at least one concomitant disease (Table 3).

### 3.2. COVID-19 Acute Infection

**Figure 2:**
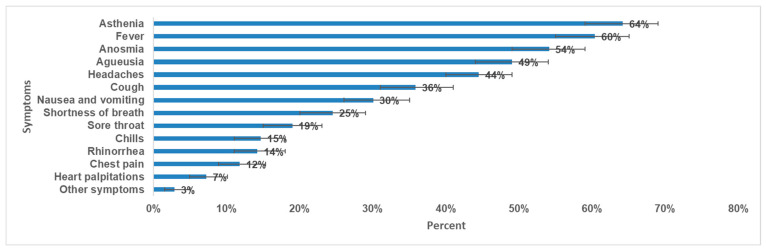
Prevalence of symptoms during SARS-CoV-2 acute infection and 95% CI, long-COVID cross-sectional study in Tunisia, 2020/2021 (*n* = 416).

Less than half (49%) of participants (204 out of 416) were confirmed to have COVID-19 during the circulation of wild strain and 26% (109 out of 416) were confirmed during the circulation of SARS-CoV-2 delta variant predominance. During the SARS-CoV-2 acute infection, 90% (373 out of 416) were symptomatic, 56,0% (*n* = 233) experienced an intense form, and 17% (*n* = 71) experienced a severe form. The most prevalent symptoms were asthenia (64%), fever (60%), anosmia (54%), and ageusia (49%) (Figure 2). Eighty-six per cent (*n* = 358) were vaccinated against COVID-19 (at least one dose) and 72% (*n* = 300) received treatment during the SARS-CoV-2 acute infection.

### 3.3. Long-COVID Prevalence and Characteristics

**Figure 3:**
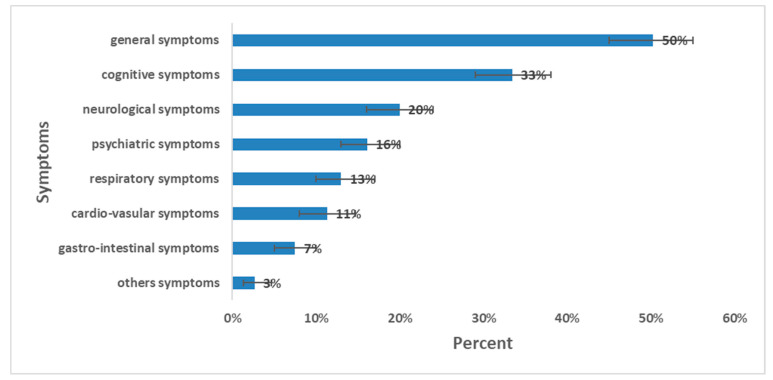
Prevalence of long-COVID symptoms and 95% CI, long-COVID cross-sectional study, Tunisia, 2020/2021 (*n* = 416).

The prevalence of long COVID was 64% (267 out of 416); 95% CI [59–69%]. The sex ratio of long-COVID cases (M:F) was 0.72. The prevalence of long COVID among females was 73%; (95% CI [67–79%]). By age group, the highest prevalence was observed among those aged 18–64 years with a prevalence of 68%; 95% CI [63–73%] (Table 4). The most prevalent symptoms were general symptoms (50%); mainly fatigue (32%), and myalgia/arthralgia (17%) followed by cognitive symptoms (33%) dominated by memory loss (27%) and concentration problems (17%) (Figure 3). The median duration of long-COVID symptoms was 11 months (IQR: 3–14 months).

### 3.4. Long-COVID Determinants

Compared with respondents under 18 years old, those aged 18–64 had a higher prevalence of long COVID (PR = 2.3; 95% CI [1.3–4.0]). In addition, we estimated higher prevalence of long-COVID among females (PR = 1.3; 95% CI [1.1–1.5]), women of childbearing age (PR = 1.3; 95% CI [1.1–1.6]), those residing in the Center region (PR = 1.7; 95% CI [1.3–2.2]) compared to those residing in the southern region and among healthcare workers (PR = 1.2; 95% CI [1.0–1.5]). Being symptomatic during acute infection (PR = 2.4; 95% CI [1.5–3.9]), having intense COVID-19 (four or more symptoms during SARS-CoV-2 acute infection) (PR = 1.6; 95% CI [1.4–1.9]), experiencing severe COVID-19 (PR = 1.3; 95% CI [1.2–1.6]), being vaccinated against COVID-19 (PR = 1.6; 95% CI [1.2–2.2]), and having received specific treatment during SARS-CoV-2 acute infection (PR = 1.4; 95% CI [1.1–1.7]) were also significantly associated with the long-COVID prevalence (Table 5).

In multivariable analysis, after adjusting for confounding factors, having intense COVID-19 (four or more symptoms during SARS-CoV-2 acute infection), residing in the Center region, and being a woman of childbearing age were independently associated with a higher prevalence of long COVID (Table 6).

## 4. Discussion

We estimated the prevalence of long COVID among the Tunisian population. To recruit a representative sample, we stratified the source population by age and residence to adjust for those potential confounding factors. Long-COVID prevalence was 64%; 95% CI: [59–69%]. Patients with long-COVID symptoms were mostly female and aged between 18 and 64 years. The most common symptoms were general symptoms followed by cognitive symptoms. However, these results should be interpreted with caution due to some limitations.

Firstly, the response rate was low (38%), potentially leading to a selection bias. However, to assess the possible impact of non-response, we made two attempts to contact non-respondents. Also, a non-response analysis showed non-significant difference in key demographic variable between respondents and non-respondents. Indeed, the observed sample size (*n* = 416) was also higher than the expected sample size (*n* = 384). Secondly, long COVID was self-reported by participants without any objective clinical diagnosis which could introduce misclassification and therefore overestimate the long-COVID prevalence. However, given the absence of universally accepted diagnostic criteria for long COVID, the use of sensitive and arbitrary criteria remains necessary. Although the case definition applied in this study was adapted from international standards (WHO and CDC), its evolving nature may have influenced the estimated prevalence and identified determinants of long COVID. Thirdly, the cross-sectional study design did not allow for assessing temporality between exposures and outcome and consequently, the causality between exposures and long COVID. Also, some people could develop long-COVID symptoms without being tested for COVID-19, potentially leading to an underestimation of long-COVID prevalence. However, the national testing strategy and capacity allowed for the majority of eligible people to be screened.

In addition, there were some limitations associated with the telephone interview. We could not assess the influence of socio-economic status which had previously shown association with long-COVID symptoms. The vaccination status and vaccination date were self-reported by participants since no vaccination card was available. This could potentially introduce recall bias and consequently lead to an underestimation of the true impact of COVID-19 vaccines on long COVID.

Furthermore, respondents might not accurately recall the exact duration of long-COVID symptoms; however, having persistent symptoms after SARS-CoV-2 acute infection should be memorable. Finally, we did not use individual sequencing data for SARS-CoV-2 variant circulation period given the limited sequencing activity compared to eligible specimens. In fact, we relied mainly on the calendar period of dominant SARS-CoV-2 VOC circulation which was performed among representative samples according to the national sequencing strategy [17]. The ecological nature of that attribution could affect the long-COVID prevalence by period.

Interviewing children through their legal guardian could not be a good alternative but including this sub-population makes the prevalence of long COVID more representative of the general population. For children aged 12–18 years, the interview was conducted in the presence of the legal guardian.

Finally, we did not validate the questionnaire before data collection. Some long COVID studies highlighted the effectiveness of validate questionnaire in measuring the symptoms and impact of long COVID [19]. To mitigate the lack of formal prior validation, the questionnaire was pilot-tested, reviewed by experts, and administered by trained interviewers using standardized procedures.

### 4.1. Long-COVID Prevalence

At the national level, the prevalence found in our study after consideration of the methodological variations was in the range previously estimated: 77.4%, 64.1% and 46.5% [10,11,12]. Long-COVID prevalence varies widely worldwide ranging from 4.7% to 80% which can be attributed to the differences between the studied populations, study design (prospective or cross sectional), the observation periods, the criteria used to define long COVID, as well as the timeframe and accuracy of self-reporting symptoms [20,21]. In the United Kingdom, as of September 2022, 3.3% of the population (estimated 2.1 million people) self-reported experiencing long-COVID symptoms after the first confirmed or suspected SARS-CoV-2 infection [22]. In France, a study conducted in March-April 2022 using an online self-administered questionnaire reported a long-COVID prevalence of 4% among participants, with 30% of them indicating a history of reported SARS-CoV-2 infection [23].

### 4.2. Long-COVID Symptoms

In our study, long-COVID symptoms were mainly physical and differed from the acute SARS-CoV-2 infection since general symptoms, especially fatigue, were the most frequently reported symptom among participants with long COVID (63%). Compared to the other national studies, the three most frequently reported symptoms were fatigue (42%), shortness of breath (41%), and headaches (22%) for the TUN-EndCOV study, breathlessness (50%), memory disorders (39%), and asthenia (38%) for the study among discharged patients from the emergency unit of Ariana Mami University hospital and fatigue (64%), memory disturbance (61%), and concentration difficulties (49%) for the online self-administered study among the Tunisian population [10,11,12]. Given that the clinical manifestations of long COVID are extremely variable, there is no specific framework that defines their characteristics; in fact, long-COVID symptoms are generally divided into general and organ-specific manifestations [24]. In the existing literature, non-respiratory symptoms, particularly fatigue, were also the most commonly reported symptoms for participants with long COVID [10,21,22,25,26,27,28]. According to a recent study conducted in Italy between February and April 2025 among 250 participants, persistent symptoms were excessive tiredness, weakness, and muscle/joint pain [29], It is interesting to note that post-viral fatigue was also reported in previous viral epidemics and pandemics including the Spanish flu in 1918, the SARS-CoV-1 virus in 2003, influenza A(H1N1) in 2009, the West Nile virus in 2012, and the Ebolavirus epidemic in West Africa in 2014 and 2016. Some of them were qualified as Myalgic Encephalomyelitis/Chronic Fatigue Syndrome (ME/CFS) [30]. Similar symptoms have been observed following other viral infections such as the Epstein–Barr virus, mononucleosis, and dengue as well as non-viral infections such as Q fever, Lyme disease, and giardiasis [30]. According to our findings, the median duration of long-COVID symptoms was 11 months. Persistent long-COVID symptoms were not reported until 12 months, even 2 years, after the acute phase [31]. More than 200 symptoms have been identified with impacts on multiple bodily systems [32].

### 4.3. Factors Associated with Long-COVID Prevalence

Our results highlighted that having multiple symptoms during SARS-CoV-2 acute infection (at least four symptoms) (a PR = 1.5), being a woman of childbearing age (a PR = 1.2), and residence in the central region (a PR = 1.5) were significantly associated with higher long-COVID prevalence in multivariable analysis. These findings can be explained by various factors, including the central region’s role as the epi-center of the epidemic, marked by the highest rates of infection and mortality during the circulation of Delta SARS-CoV-2 variant. Care access was also affected by SARS-CoV-2 variants’ circulation. During this critical period, the health-care system was overloaded, potentially impacting the management of COVID-19 cases. In fact, a higher viral load was reported for the delta variant compared to the historical and alpha variants which could explain the higher risk of developing long COVID [33,34]. Young women tend to be more attentive to their bodies and related distress; thus, they are more likely to report persistent symptoms (reporting bias). In addition, different factors could explain the higher prevalence among females including the hormonal role in perturbation of the hyperinflammatory status and a stronger IgG antibodies production in females during the early phase of diseases. Also, an important number of women are employed within the healthcare systems, leading to increased exposure to COVID-19 patients [35]. In our study, there was no significant association between COVID-19 vaccination and long COVID in multivariable analysis. COVID-19 vaccination by protecting mainly from COVID-19 severe outcomes like hospitalization and death, reduces significantly morbidity and mortality of the disease. It might have protective and therapeutic effects on long COVID [12,36,37,38]. Other studies indicate that COVID-19 vaccines provide partial protection, with a reduced risk of long COVID ranging between 15% and 41% [39,40].

However, precisely quantifying this effect (before or after acute infection) remains a challenge in observational data and the conduct of clinical trials are needed [41]. Our results also showed that long COVID was not associated with SARS-CoV-2 variants. According to a systematic literature review, prevalence of long COVID was higher in individuals infected with the wild variant (50%) compared to those infected with the Alpha, Delta, or Omicron variants [42]. Another systematic review and meta-analysis found that pooled long-COVID prevalence was the highest during the alpha variant 65.8% (95% CI 47.7%, 83.9%) followed by wild-type variant: 52.1% (95% CI: 44.0%, 60.1%) [36]. Comparing our findings with other national studies, female gender was an associated factor to long COVID in addition to endothelial dysfunction (endothelial quality index EQI < 2) and severe clinical status during acute COVID-19 according to the TUN-EndCOV study and Ariana study [10,37]. While for the online study among the Tunisian population, the associated factors were female gender and age of 60 years or older and complete COVID-19 vaccination [12]. However, tobacco consumption, severity of SARS-CoV-2 acute infection, and pre-existing conditions including obesity, diabetes, asthma, and cardiovascular disease were not significantly associated with the long-COVID prevalence in our study. Overall, our results regarding the determinants of long COVID are aligned with the existing literature suggesting that long-COVID affects COVID-19 survivors across all levels of disease severity including asymptomatic, mild, and those not tested for COVID-19. This impact is observed among diverse groups including young adults, children, and those who were not hospitalized [38].

## 5. Conclusions

Long COVID is prevalent in Tunisia, particularly among young women, patients with multiple symptoms during the acute phase of SARS-CoV-2 infection, and residents of the central region. Interpretation of these findings is limited by potential recall bias in symptom duration, self-reported vaccination status, and the inherent limitations of a cross-sectional design.

Given the novelty of this condition, further research is needed to improve its prevention, diagnosis, treatment, and care. Strengthening health education and communication throughout all stages of COVID-19 is essential to promote early medical consultation for persistent symptoms and to emphasize preventive measures, including vaccination, non-pharmaceutical interventions, and celebration of the international Long-COVID Awareness day on 15 March each year. Improving healthcare access and follow-up, especially for high-risk groups, along with raising awareness among policymakers, may contribute to better management of long COVID in Tunisia.

## Figures and Tables

**Table 1:** The minimum number of participants to be recruited by stratum, long-COVID cross-sectional study in Tunisia, 2020–2021 (*n*_sample_ = 768 among *N* = 479,743).

Region of Residence	North	Center	South
**-Number (percentage)**	204,468 (43%)	179,186 (37%)	96,089 (20%)
**-Sample size per region**	43% × 68 = 330	37% × 768 = 284	20% × 768 = 154
**-Sample size per age groups in each region**			
**0–17**	6% × 330 = 20	6% × 284 = 18	7% × 154 = 11
**18–64**	80% × 330 = 263	80% × 284 = 228	78% × 154 = 120
**≥65**	10% × 330 = 34	11% × 284 = 32	13% × 154 = 20

**Table 2:** Non-response analysis, long-COVID cross-sectional study, Tunisia, 2020–2021 (*n* = 1095).

Characteristics	Respondents (*n* = 416)	Non-Respondents (*n* = 679)	*p*-Value
**Gender**			0.14
Male	204	302	
Female	212	377	
**Age group (years)**			
0–17	30	45	0.09
18–64	345	537	
≥65	41	97	
**Region of residence**			0.6
North	181	302	
Center	157	265	
South	78	112	
**Period of COVID-19 confirmation**			0.6
P1 (September 2020–February 2021)	219	347	
P2 (March–September 2021)	197	332	

**Table 3:** Characteristics of the study population, long-COVID cross-sectional study, Tunisia, 2020–2021 (*n* = 416).

Characteristics		Number	Percentages (%)
**Gender**	Male	204	49
	Female	212	51
**Age group (years)**	0–17	30	7
	18–64	345	83
	≥65	41	10
**Women of childbearing age (15–49 years)**	Yes	139	33
	No NA	73 204	18 49
**Region of residence**	North	181	43
	Center	157	38
	South	78	19
**Obesity (BMI ≥ 30 Kg/m^2^)**	Yes	96	23
	No	305	73
	NP/NA	15	4
**Education level**	Less than secondary level	96	23
	At least secondary level	307	74
	NP/NA	13	3
**Healthcare workers**	Yes	44	11
	No	301	72
	NP/NA	71	17
**At least one concomitant disease**	Yes	135	32
	No	281	68
**Tobacco consumption**	Yes	80	19
	No	323	78
	NP/NA	13	3
**Alcohol consumption**	Yes	28	7
	No	371	89
	NP/NA	17	4
**Sedentary lifestyle**	Yes	190	46
	No	211	51
	NP/NA	15	3

NP/NA: Not precise, not applicable.

**Table 4:** Long-COVID prevalence by main socio-demographic characteristics, long-COVID cross-sectional study, Tunisia, 2020–2021 (*n* = 416).

Characteristics	Number of Long-COVID Cases (*n* = 267)	Study Population (*n* = 416)	Prevalence; 95% CI
**Age group (years)**			
0–17	9	30	30%; 15%–49%
18–64	236	345	68%; 63%–73%
≥65	22	41	54%; 37%–69%
**Gender**			
Male	112	204	55%; 48%–62%
Female	155	212	73%; 67%–79%
**Region of residence**			
North	115	181	64%; 56%–71%
Center	118	157	75%; 68%–82%
South	34	78	44%; 32%–55%

CI: confidence interval.

**Table 5:** Factors associated with long COVID in univariable analysis, long-COVID cross- sectional study in Tunisia, 2020–2021 (*n* = 416).

Exposures		Crude PR	95% CI	*p*-Value
**Socio-demographic characteristics**				
Age group (years)	18–64 (*n* = 345)	2.3	1.3–4.0	**<0.001**
	≥65 (*n* = 41)	1.8	1.0–3.3	
	0–17 (*n* = 30)	Ref		
**Gender**	Female (*n* = 212)	1.3	1.1–1.5	**<0.001**
	Male (*n* = 204)	Ref		
**Being a woman of childbearing age (15–49 years) ⸉ (*n* = 212)**	Yes (*n* = 139)	1.3	1.1–1.6	**0.006**
	No (*n* = 73)	Ref		
**Region of residence**	North (*n* = 181)	1.4	1.1–1.9	**<0.001**
	Center (*n* = 157)	1.7	1.3–2.2	
	South (*n* = 78)	Ref		
**Education level**	Less than secondary level (*n* = 96)	1.1	0.9–1.3	0.4
	At least secondary level (*n* = 307)	Ref		
**Healthcare worker**	Yes (*n* = 44)	1.2	1.0–1.5	**0.048**
	No (*n* = 301)	Ref		
**Comorbidities**	Yes (*n* = 135)	1.1	0.9–1.3	0.2
	No (*n* = 281)	Ref		
**Lifestyle**				
Obesity (BMI ≥ 30 Kg/m^2^)	Yes (*n* = 96)	1.0	0.8–1.2	0.76
	No (*n* = 305)	Ref		
Tobacco consumption	Yes (*n* = 80)	0.9	0.8–1.2	0.6
	No (*n* = 323)	Ref		
Alcohol consumption	Yes (*n* = 28)	1.0	0.7–1.3	1.0
	No (*n* = 371)	Ref		
Sedentary lifestyle	Yes (*n* = 190)	1.1	0.9–1.2	0.5
	No (*n* = 211)	Ref		
	No (*n* = 281)	Ref		
**SARS-CoV-2 acute infection**				
Pregnancy or post-partum during SARS-CoV-2 infection ⸉	Yes (*n* = 12) No (*n* = 105)	1.2 Ref	1.0–1.5	0.2
**Period of SARS-CoV-2 infection confirmation**	Alpha variant (*n* = 103)	1.1	0.9–1.3	0.6
	Delta variant (*n* = 109)	1.0	0.8–1.2	
	Wild variant (*n* = 204)	Ref		
**Symptoms during SARS-CoV-2 acute infection**	Yes (*n* = 370)	2.4	1.5–3.9	**<0.001**
	No (*n* = 46)	Ref		
**Intense COVID-19**	≥4 (*n* = 233)	1.6	1.4–1.9	**<0.001**
	<4 (*n* = 183)	Ref		
**Severe COVID-19**	Yes (*n* = 71)	1.3	1.2–1.6	**0.001**
	No (*n* = 345)	Ref		
**SARS-CoV-2 re-infection**	Yes (*n* = 62)	1.1	0.9–1.3	0.2
	No (*n* = 354)	Ref		
COVID-19 vaccination status *	Vaccinated (*n* = 359)	1.6	1.2–2.2	**0.001**
	Unvaccinated (*n* = 57)	Ref		
COVID-19 treatment **	Yes (*n* = 300)	1.4	1.1–1.7	**<0.001**
	No (*n* = 116)	Ref		

⸉: Calculated only if gender = female (*n* = 212); * At least one dose; **: corticoids, chloroquine, antibiotics, anti-coagulants; PR = prevalence ratio; CI: confidence interval. Bold ***p*-Value**—significant values of ***p*** (<0.05).

**Table 6:** Factors associated with long COVID in log-binomial regression (multivariable analysis), long-COVID cross-sectional study, Tunisia, 2020–2021 (*n* = 196).

Exposures	Adjusted PR	95% CI	*p*-Value
**Intense COVID-19** (≥4 symptoms)	1.5	1.0–2.1	**0.04**
**Region of residence** Center North	1.5 1.3	1.1–2.0 1.0–1.8	**0.005**
**Being a woman of childbearing age** (15–49 years)	1.2	1.0–1.4	**0.038**

Akaike information criterion (AIC) = 197.8; PR = prevalence ratio; CI: confidence interval. Bold ***p*-Value**—significant values of ***p*** (< 0.05).

## Data Availability

Data will be available upon request.

## Supplementary Materials

The following supporting information can be downloaded at https://www.mdpi.com/article/10.3390/ijerph23010049/s1, Supplementary Materials S1: Questionnaire: Long-term health effects of COVID-19 in Tunisia, 2020/2021, Supplementary Materials S2: ROC curve for COVID-19 acute infection, long COVID cross-sectional study, Tunisia, 2020/2021 (*n* = 416), Supplementary Materials S3: Heat map for long COVID symptoms, long COVID cross-sectional study, Tunisia, 2020/2021 (*n* = 416), Supplementary Materials S4: Checking of representativeness of the study population according to the stratification criteria, long-COVID cross-sectional study, Tunisia, 2020–2021.
