# Supplementary material for: Long-Term Health Effects of COVID-19 in Tunisia, 2020–2021
Source: Int J Environ Res Public Health. 2025 Dec 30;23(1):49. doi: 10.3390/ijerph23010049 (PMC12840641; doi:10.3390/ijerph23010049)
Supplement: Supplementary file 1 [file ijerph-23-00049-s001.zip › ijerph-3962141-supplementary.pdf]

Supplementary Materials S1: Questionnaire : Long-term health effects of COVID-19 in Tunisia, 2020/2021

<u>Oral Informed consent :</u>

Date of the phone-survey: [DD/MM/Years]

Investigator name: [Consultant name]

Patient identity: [First name] [Last name], [Age]

My name is [investigator name] and I work at the National Observatory of New and Emerging Diseases, Ministry of Health, Tunis, Tunisia. You were infected by SARS-Cov-2. We call you today to monitor your heath status after COVID-19 disease.

If you agree, I will ask you some questions that will be used to orientate your needs of medical monitoring specific to your COVID-19 disease.

May I continue this interview ? [YES / NO]

If no, why? [text]

1-Consultant's infomations :

- Date of the teleconsultation: DD /MM/Years
- Consultant name:
- Consultant phone :

2-Patient's general informations :

- Identification number (ID) :
- Gender : Male ☐; Female ☐
- Date of birth : DD /MM/Years ; Age (years)
- Governorate of residence :
- Health care worker : Yes ☐ ; No ☐
- Occupation level :
  *Worker ☐
  *Middle management ☐
  *Senior ☐
  *Unemplyed ☐
  *Student ☐

- Education : Illeterate ☐
  Primary, secondary, post secondary level ☐
  Universitary level ☐

- Annual Income (TND) : <5000
  >=5000

3-Patient's clinical characteristics and life style :

- BMI (Kg/m^2) : ;Obesity : Yes ☐ ; No ☐
- Active Tobacco consumption : Yes ☐ ; No ☐
- Alcohol consumption : Yes ☐ ; No ☐
- Sedentariness : Yes ☐ ; No ☐
- Comorbidities : Yes ☐ ;No ☐

If Yes, type of comorbidities:

- High Blood Pressure (HBP) ☐
- Diabetes ☐
- lung disease : Chronic obstructive pulmonary disease ☐ ; Asthma ☐ ; Tuberculosis ☐
- Heart disease ☐
- Kidney disease ☐ Chronic dialysis ☐
- Immunopressive condition : cancer ☐ , immunodeficiency ☐ , HIV infection ☐
- Psychiatric condition ☐
- Neurogilical condition ☐
- Others……………………………
- For women : Pregnancy during acute infection : Yes ☐ ; No ☐
- If yes term of pregnancy : Trimester 1 ☐ ; Trimester 2 ☐ ; Trimester 3 ☐

4-COVID-19 related data :

- Date of COVID-19 confirmation : DD /MM/Years
- Mode of confirmation : RT-PCR ☐ , TDR-Ag ☐ , CT-Scan ☐
- Symptomatic ☐ ; Asymptomatic ☐
- **If symptomatic :**
- Date of onset of symptoms : DD /MM/Years
- Types of symptoms in acute infection :

- **General signs :**

Anorexia ☐,

Weight loss ☐

Tiredness or fatigue ☐

Joint or muscle pain ☐

Fever ☐

Rash ☐

Post-exertional malaise (Symptoms that get worse after physical or mental activities) ☐:

Changes in menstrual period cycles ☐

Hair loss ☐

Vertigo ☐

- **Respiratory symptom :**

Difficulty breathing or shortness of breath (New onset dyspnea) ☐

Wheezing ☐

New onset cough ☐

New onset allergies ☐

Abnormal lung CT-scan since discharge ☐

- **Heart symptoms :**

Tachycardia/palpitations ☐

Ortostatic hypotension ☐

Chest discomfort, Chest pain ☐

- **Neurologic signs :**

Headache ☐

Paresthesia (Pins-and-needles feeling) ☐

Change in smell or taste ☐

- **Cognitive signs :**

Memory losses ☐

Slowness for reasoning, activity planification or problem solving ☐

Concentration, attention or thinking difficulties (brain fog) ☐

Sleep problems (insomnia) ☐

Mood changes ☐ If Yes : duration (days) :

Symptoms of post-traumatic stress disorder ☐

Depression ☐

- **Gastrointestinal signs :**

Loss of appetite ☐

Diarrhoea ☐

Constipation ☐

Acid reflux ☐

- Number of symtoms first week of illness : 1 ☐ ; 2-4 ☐; >=5 ☐
- Duration of symptoms : <10 d ☐

10-28 d ☐

> 1 month ☐

> 2 months ☐

> 3 months ☐

> 6 months ☐

- Oxygenotherapie supplementation : Yes ☐ ; No ☐
- Hospitalization during acute infection phase : Yes ☐ ; No ☐

If yes : duration of hospitalization (days) : …………………

- Service of hospitalization ICU ☐ ; other COVID-19 isolation service ………………
- Duration of hospitalization in ICU : ….. days
- Complications during Actue infection : Yes ☐ ;No ☐

If yes : Type of complications :

- Pulmonary embolism ☐
- Acute respiratory failure ☐
- Acute cardiac failure ☐
- Specific treatment received in acute infection Yes ☐ ;No ☐

Severity grade of acute infection :

Asymptomatic ☐

Mild/Moderate ☐

Critical/Severe ☐

If Yes : Antibiotic (Zithromax) ☐

Hydroxychloroquine ☐

Corticosteroids ☐

Anticoagulation ☐

- Date of recovery from acute illness : DD /MM/Years
- COVID-19 vaccination : Yes ☐ ; No ☐

if yes : Name of vaccine :

Date of vaccination :

5-Long COVID-19's symptoms : For particpants who said yes in duration of symptoms >28d

- Types of symptoms :
- **General signs :**

Anorexia ☐ If Yes : duration (days) :

Weight loss ☐ If Yes : duration (days) :

Tiredness or fatigue ☐ If Yes : duration (days) :

Joint or muscle pain☐ If Yes : duration (days) :

Fever☐ If Yes : duration (days) :

Rash☐ If Yes : duration (days) :

Diarrhea☐ If Yes : duration (days) :

Post-exertional malaise (Symptoms that get worse after physical or mental activities)☐ If Yes : duration (days) :

Changes in menstrual period cycles☐ If Yes : duration (days) :

- **Respiratory symptom :**

Difficulty breathing or shortness of breath (New onset dyspnea)☐ If Yes : duration (days) :

New onset cough☐ If Yes : duration (days) :

Abnormal lung CT-scan since discharge☐ If Yes : duration (days) :

- **Heart symptoms :**

Heart palpitations☐ If Yes : duration (days) :

Ortostatic hypotension☐ If Yes : duration (days) :

Chest discomfort, Chest pain☐ If Yes : duration (days) :

- **Neurologic signs :**

Headache☐ If Yes : duration (days) :

Paresthesia (Pins-and-needles feeling)☐ If Yes : duration (days) :

Change in smell or taste☐ If Yes : duration (days) :

- **Cognitive signs :**

Memory losses☐ If Yes : duration (days) :

Slowness for reasoning, activity planification or problem solving☐ If Yes : duration (days) :

Concentration, attention or thinking difficulties (brain fog)☐ If Yes : duration (days) :

Sleep problems☐ If Yes : duration (days) :

Mood changes☐ If Yes : duration (days) :

Symptoms of post-traumatic stress disorder☐ If Yes : duration (days) :

Depression☐ If Yes : duration (days) :

- **Gastrointestinal signs :**

Loss of appetite ☐ If Yes : duration (days) :

Diarrhoea ☐ If Yes : duration (days) :

Constipation ☐ If Yes : duration (days) :

Acid reflux ☐ If Yes : duration (days) :

Supplementary Materials S2: ROC curve for COVID-19 acute infection, long COVID study, Tunisia, 2022 (*n*= 416)

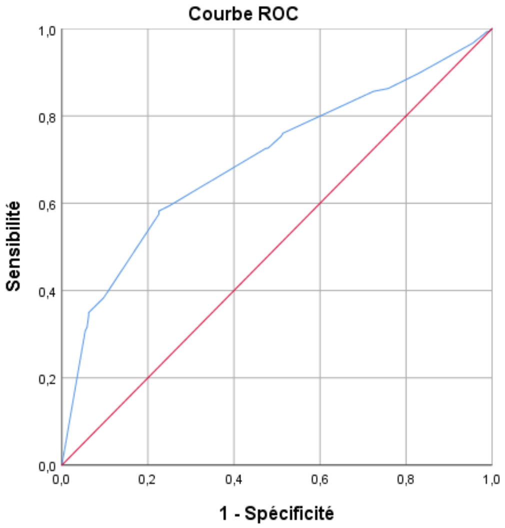

Les segments diagonaux sont générés par les liens.

Supplementary Materials S3: Heat map for long COVID symptoms, long COVID cross-sectional study, Tunisia, 2020/2021 (n= 416)

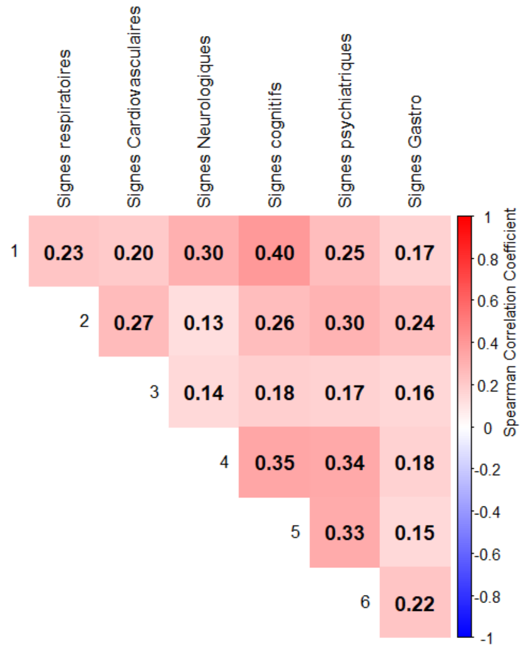

Supplementary Materials S4: Checking of representativeness of the study population according to the stratification criteria, long-COVID cross-sectional study, Tunisia, 2020–2021

Region	Source database	Study population	p-value
North	204468	181	0.8
Center	179186	157	
South	96089	78	
**Total**	**479743**	**416**	

Age group (years)	Source database	Study population	p-value
0-17	35269	30	0.4
18-64	386556	345	
65+	57917	41	
Total	479743	416	
